# Supplementary material for: Natural Antibodies Produced in Vaccinated Patients and COVID-19 Convalescents Recognize and Hydrolyze Oligopeptides Corresponding to the S-Protein of SARS-CoV-2
Source: Vaccines (Basel). 2023 Sep 15;11(9):1494. doi: 10.3390/vaccines11091494 (PMC10535122; doi:10.3390/vaccines11091494)
Supplement: Supplementary file 1 [file vaccines-11-01494-s001.zip › vaccines-2501713-supplementary.pdf]

vaccines

MDPI

Supplementary data

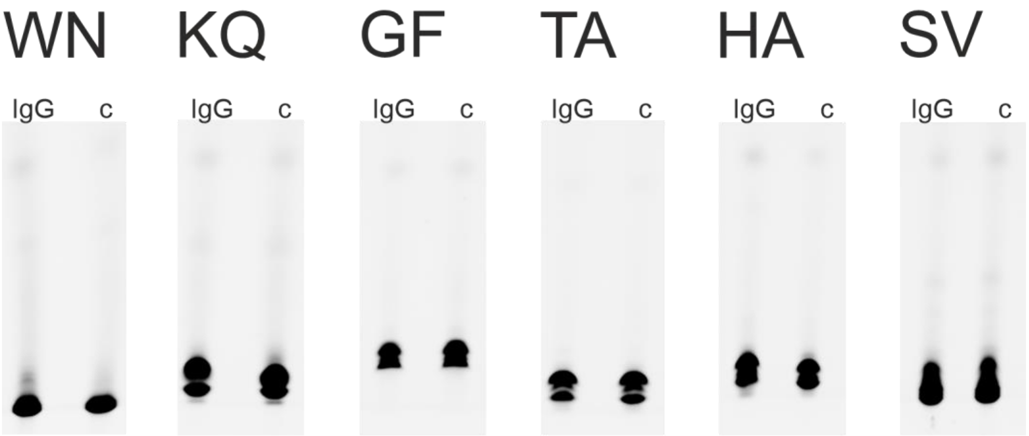

Figure S1. Analysis of OPs hydrolysis by antibodies from donors of the control group (which did not have COVID-19 and were not vaccinated). C - control without antibodies.

Table S1. Anonymized data on patients involved in the study.

**Con+Vac**							
ID	Gender	Age	Date of blood collection	Date of I Vaccination	Date of II Vaccination	Antibody titer to S-protein, PC	Antibody titer to N-protein, PC
2024	M	57	18.02.2021	14.01.2021	04.02.2021	8.8	6.2
2032	F	40	26.02.2021	01.02.2021	22.02.2021	11.1	5.8
2033	F	39	26.02.2021	09.02.2021	02.03.2021	11.3	9.6
2034	F	22	26.02.2021	09.02.2021	02.03.2021	10.8	5.4
2103	F	29	18.06.2021	26.04.2021	17.05.2021	14.1	7.3
2105	F	28	23.06.2021	08.02.2021	02.03.2021	14.3	8.5
2112	F	40	30.06.2021	08.02.2021	01.03.2021	8.6	5.1
2128	F	22	16.08.2021	22.02.2021	24.03.2021	8.2	6.9
2132	F	42	23.08.2021	08.02.2021	01.03.2021	9.6	7.2
2131	F	38	23.08.2021	08.02.2021	01.03.2021	10.3	8.5
2134	M	56	23.08.2021	08.02.2021	01.03.2021	9.7	7.9
2145	F	38	15.09.2021	25.12.2021	15.01.2021	11.0	10.1
2146	M	35	15.09.2021	08.02.2021	01.03.2021	6.1	3.3
2158	M	34	07.10.2021	08.02.2021	01.03.2021	12.4	8.2
2178	F	65	20.10.2021	08.02.2021	01.03.2021	7.7	7.6
2203	M	32	08.02.2022	09.02.2021	02.03.2021	13.2	7.4
2204	F	35	08.02.2022	09.02.2021	02.03.2021	4.2	9.1
2214	M	30	24.02.2022	01.03.2021	21.03.2021	5.8	8.3
2215	F	19	24.02.2022	01.09.2021	22.09.2021	7.8	6.6
2096	F	33	07.06.2021	08.04.2021	30.04.2021	12.1	5.2
2097	M	33	07.06.2021	08.04.2021	30.04.2021	12.5	3.7
2142	F	41	13.09.2021	08.02.2021	01.03.2021	9.8	4.2
2148	F	36	16.09.2021	09.08.2021	30.08.2021	6.6	5.3
2149	F	57	16.09.2021	09.08.2021	30.08.2021	5.1	6.5
2150	M	36	20.09.2021	06.08.2021	27.08.2021	12.8	7.4
Statistics							
Mean ± SD		37±11				9.8±2.8	6.9±1.8
Median [Q1; Q3]; Min–Max		36 [32; 40] 19-65				9.8 [7.8; 12.1] 4.2-14.3	7.2 [5.4; 8.2] 3.3-10.1

**Con**						
ID	Gender	Age	Date of blood collection	Period after the first symptoms of COVID-19, weeks	Antibody titer to S-protein, PC	Antibody titer to N-protein, PC

1016	M	42	09.11.2020	3	10.0	6.3
1020	F	35	18.11.2020	6	7.4	5.2
1026	M	21	19.11.2020	5	7.8	5.4
1040	M	36	30.11.2020	6	11.0	7.8
1091	M	36	23.12.2020	10	13.4	10.9
1116	F	66	12.01.2021	6	8.5	6.8
1117	M	75	12.01.2021	6	9.2	5.3
1131	F	35	15.01.2021	6	14.4	10.7
1132	F	26	15.01.2021	6	8.1	6.4
1136	F	28	14.01.2021	8	9.4	7.9
1137	M	24	14.01.2021	12	11.5	9.8
1138	M	42	18.01.2021	14	10.8	8.8
1140	F	32	19.01.2021	9	7.2	4.5
1142	F	38	14.01.2021	6	12.9	10.5
1143	F	37	19.01.2021	6,5	12.8	9.6
1145	M	36	21.01.2021	14	12.9	10.8
1146	F	75	21.01.2021	8	11.5	9.3
1156	M	45	28.01.2021	12	7.2	3.2
1159	F	62	28.01.2021	16	8.5	5.1
1164	F	24	28.01.2021	6	9.1	5.9
1167	F	59	29.01.2021	13	8.4	4.7
1176	F	42	01.02.2021	13	8.0	3.4
1180	F	37	02.02.2021	7	7.2	4.6
1206	M	36	25.02.2021	15	11.0	8.5
1212	M	57	01.03.2021	13	13.2	9.1
Statistics						
Mean ± SD		42±15			10.1±2.3	7.2±2.5
Median [Q1; Q3]; Min–Max		37 [35; 45]<br>21-75			9.4 [8.1; 11.5]<br>7.2-14.4	6.8 [5.2; 9.3]<br>3.2-10.9

Vac

ID	Gender	Age	Date of blood collection	Date of I vaccination	Date of II vaccination	Antibody titer to S-protein, PC	Antibody titer to N-protein, PC
2011	F	38	03.02.2021	11.01.2021	14.01.2021	11.0	0.3
2012	F	45	03.02.2021	24.12.2020	14.01.2021	12.1	0.2
2013	F	41	03.02.2021	24.12.2020	24.12.2020	11.2	0.2
2017	F	40	08.02.2021	27.12.2020	20.01.2021	11.6	0.2

2018	M	29	08.02.2021	27.12.2020	20.01.2021	11.4	0.1
2019	F	42	08.02.2021	03.12.2020	24.12.2020	11.5	0.2
2020	M	32	09.02.2021	29.12.2020	20.01.2021	13.4	0.2
2021	M	45	09.02.2021	24.12.2020	18.01.2021	11.1	0.2
2022	F	63	17.02.2021	01.12.2020	21.12.2020	6.3	0.2
2025	F	35	24.02.2021	11.01.2021	01.02.2021	9.8	0.1
2026	M	35	24.02.2021	11.01.2021	01.02.2021	6.8	0.1
2027	F	35	25.02.2021	11.01.2021	01.02.2021	9.5	0.2
2030	M	29	25.02.2021	11.01.2021	01.02.2021	9.9	0.3
2035	M	30	26.02.2021	11.01.2021	01.02.2021	10.7	0.2
2036	M	50	26.02.2021	11.01.2021	01.02.2021	8.6	0.2
2037	M	65	26.02.2021	11.01.2021	01.02.2021	8.0	0.1
2038	F	65	26.02.2021	11.01.2021	01.02.2021	8.0	0.1
2040	F	35	01.03.2021	11.01.2021	01.02.2021	8.7	0.2
2041	F	27	02.03.2021	11.01.2021	01.02.2021	8.1	0.2
2042	M	55	02.03.2021	11.01.2021	01.02.2021	8.1	0.1
2062	F	70	02.03.2021	11.01.2021	01.02.2021	8.1	0.1
2067	M	58	29.03.2021	08.02.2021	01.03.2021	6.9	0.2
2068	M	34	29.03.2021	08.02.2021	01.03.2021	6.2	0.2
2070	F	31	30.03.2021	08.02.2021	01.03.2021	11.0	0.2
2071	F	41	02.04.2021	24.12.2020	14.01.2020	11.6	0.2
Statistics							
Mean ± SD		43±13				9.6±2.0	0.2±0.1
Median [Q1; Q3]; Min–Max		40 [34; 50] 27-70				9.8 [8.1; 11.2] 6.2-13.4	0.2 [0.1; 0.2] 0.1-0.3

**Neg**					
ID	Gender	Age	Date of blood collection	Antibody titer to S-protein, PC	Antibody titer to N-protein, PC
1021	F	44	19.11.1020	0.5	0.1
1022	M	56	19.11.1020	0.3	0.1
1027	F	40	19.11.1020	0.4	0.3
1028	F	40	19.11.1020	0.2	0.2
1034	F	39	20.11.1020	0.2	0.2
1037	F	29	23.11.2020	0.3	0.2
1038	F	40	25.11.2020	0.5	0.1
1039	M	39	27.11.2020	0.8	0.1

1041	F	22	24.11.2020	0.3	0.3
1042	F	60	24.11.2020	0.3	0.2
1043	F	62	25.11.2020	0.2	0.3
1044	F	46	24.11.2020	0.2	0.1
1046	F	42	26.11.2020	0.6	0.4
1047	M	23	26.11.2020	0.3	0.2
1050	F	32	04.12.2020	0.6	0.2
1051	F	50	04.12.2020	0.1	0.2
1056	F	34	03.12.2020	0.1	0.2
1058	F	26	03.12.2020	0.2	0.1
1059	F	33	03.12.2020	0.1	0.1
1061	M	30	03.12.2020	0.6	0.2
1062	F	22	03.12.2020	0.2	0.2
1067	M	31	07.12.2020	0.3	0.2
1069	M	49	07.12.2020	0.4	0.2
1075	F	39	14.12.2020	0,2	0.3
1096	M	59	23.12.2020	0.3	0.1
Statistics					
Mean ± SD		39±12		0.3±0.2	0.2±0.1
Median [Q1; Q3]; Min–Max		39 [31; 46] 22-62		0.3 [0.2; 0.4] 0.1-0.8	0.2 [0.1; 0.2] 0.1-0.4
